# Identification of subtype-specific genes signature by WGCNA for prognostic prediction in diffuse type gastric cancer

**DOI:** 10.18632/aging.103743

**Published:** 2020-09-11

**Authors:** Qi Zhou, Li-Qiang Zhou, Shi-Hao Li, Yi-Wu Yuan, Li Liu, Jin-Liang Wang, Deng-Zhong Wu, You Wu, Lin Xin

**Affiliations:** 1 Department of General Surgery, The Second Affiliated Hospital of Nanchang University, Nanchang, Jiangxi Province, China

**Keywords:** gastric cancer, diffuse type, progression, weighted gene co-expression network analysis, signature

## Abstract

Background: Gastric cancer is a common malignancy and had poor response to treatment due to its strong heterogeneity. This study aimed to identify essential genes associated with diffuse type gastric cancer and construct a powerful prognostic model.

Results: We conducted a weighted gene co-expression network analysis (WGCN) using transcripts per million (TPM) expression data from The Cancer Genome Atlas (TCGA) to find out the module related with diffuse type gastric cancer. Combining Least Absolute Shrinkage and Selection Operator (LASSO) with multi-cox regression, the 10 specific genes risk score model of diffuse type gastric cancer was established. The concordance index (0.97), the area under the respective ROC curves (AUCs) (1-years: 0.98; 3-years: 1; 5-years: 1) and survival difference of high- and low risk groups (p=2.84e-10) of this model in TCGA dataset were obtained. The moderate predicting performance was observed in the independent cohort of GSE15459 and GSE62254. The results of the gene set enrichment analysis (GSEA) using high-and low risk group as phenotype indicated differential expression of tumor-related pathways.

Conclusion: Thus, we constructed a reliable prognostic model for diffuse type gastric cancer, which should be beneficial for clinical therapeutic decision-making.

## INTRODUCTION

Gastric cancer (GC) is the sixth most common cancer and the second leading cause of cancer-related deaths worldwide. In 2018, 1,033,701 people were diagnosed and 782,685 people died from GC [1]. Patients with GC usually have an unfavorable prognosis, as the majority reach the advanced stages of disease prior to diagnosis [2]. The lack of precision treatment and evaluation strategies have prompted researchers to investigate carcinogenic abnormalities of GC to assess survival rates and guide medical decisions. Identifying therapeutic targets and prognostic biomarkers for early GC and developing appropriate therapeutic methods are a prospective method to identify the subtypes of GC and improve the prognosis of patients with advanced gastric cancer. However, the underlying heterogeneity and complexity of GC make it difficult to identify reliable factors for effective clinical treatment [3, 4].

GC has many classification systems, such as: the Lauren classification system, and the World Health Organization (WHO) classification systems [5, 6]. Lauren classification mainly includes intestinal type and diffuse type. Because of its strong perceiving of histology and biology of gastric cancer, it has been widely used in clinical practice [7]. Intestinal type GC cells are tubular or glandular, more densely arranged and more cohesive, whereas diffuse type GC cells are usually diffuse and have poor adhesion, resulting in less glandular formation and easier diffusion. The two histological types differ in their clinical and molecular features to the point of representing distinct entities [8]. Diffuse type GC usually had characteristic mutations in genes that participate in adhesion, chromatin integrity, or cell motility [7]. Intestinal type GC exhibited aneuploidy or other genetic features more frequently.

In the past, it was not accurate to analyze GC as a whole. In this study, we divided the samples from The Cancer Genome Atlas (TCGA; https://cancergenome.nih.gov/) database to diffuse- and intestinal- type GC. we identified genes significantly associated with diffuse type based on weighted gene co-expression network analysis (WGCNA). Ten genes were obtained to construct the predicting system, which was proved an effective prognostic system for diffuse type GC.

## RESULTS

### Detection of gene co-expression modules correlated with diffuse type GC cohort

**Figure 1:**
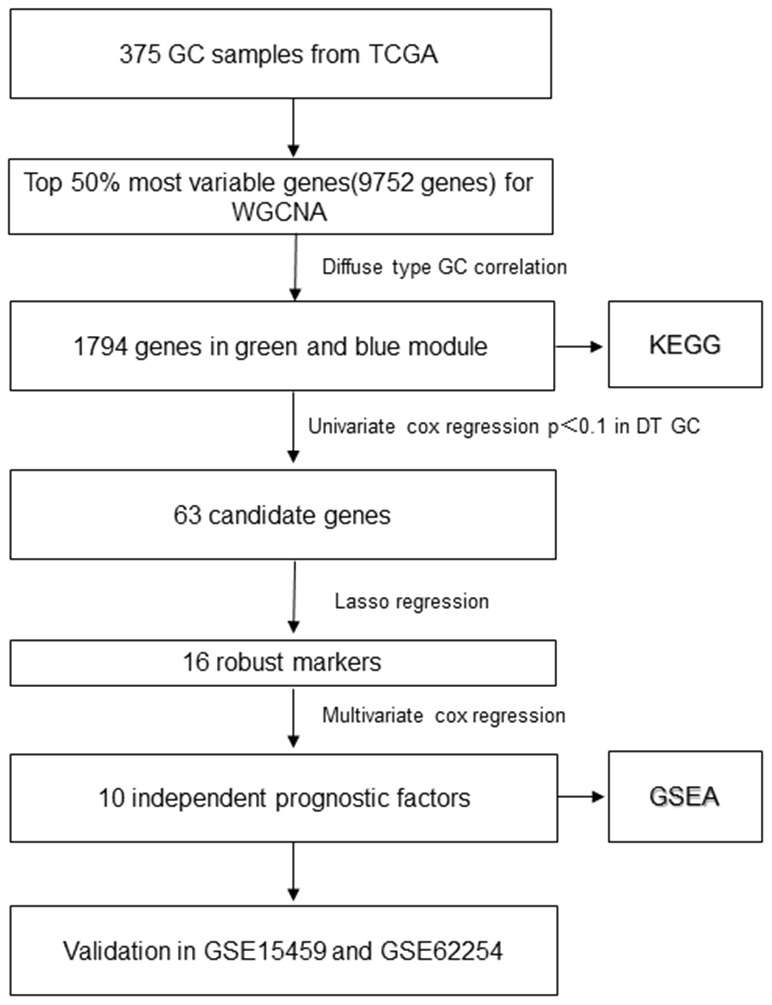
**The flowchart of identifying procedure for the multi-gene signatures in diffuse type GC.**

**Figure 2:**
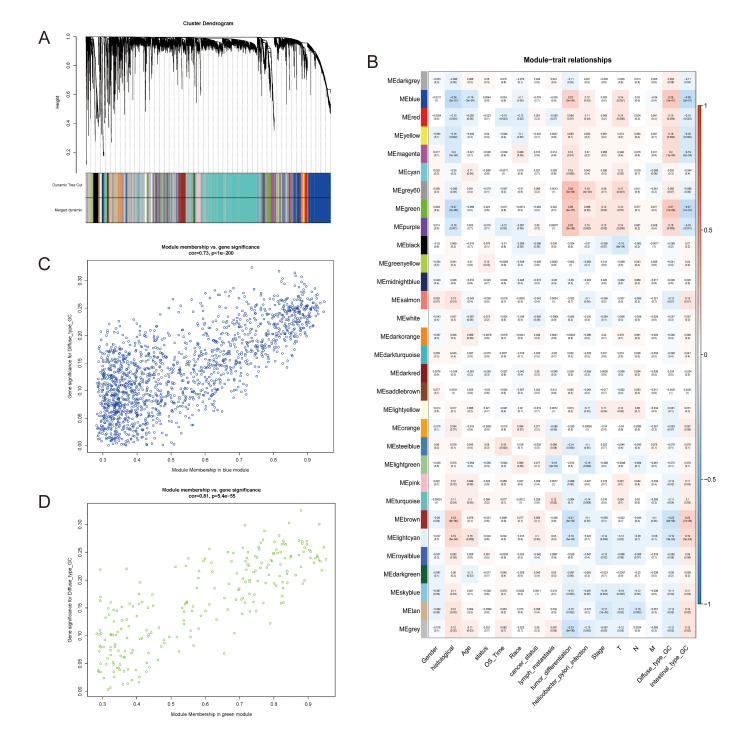
**Identification of modules associated with the diffuse type GC.** (**A**) Dendrogram of 9752 genes clustered based on a dissimilarity measure (1-TOM). (**B**) Correlation of module eigengenes with all traits. Each unit contains the corresponding correlation coefficient and P-value. The table is color-coded by correlation according to the color legend. (**C**, **D**) Scatter diagrams between blue and green modules and diffuse type GC.

The data was processed and analyzed by following the workflow in Figure 1. Top 50% most variable genes (9752 genes) were used for WGCNA. An obvious outlier was removed (Supplementary Figure 1A) and a soft threshold = 4 was selected to construct a scale-free network (Supplementary Figure 1B, 1C). A total of 30 gene modules were identified after setting the minimum cluster size as 30 (Figure 2A). The grey module contained genes not attributed to any modules. The identified genes associated with a clinical trait were of great value in the exploration of the molecular characteristics of that trait. In the present study, the clinical parameters of diffuse type GC patients, including age at diagnosis, gender, race, tumor differentiation, pathological stage, pathological T category, pathological N category, M category, lymphatic metastasis, cancer status, helicobacter pylori infection, status and overall survival (OS) time was extracted for analysis. we found that blue module (R=0.26, p=3e-07) and green module (R=0.27, p=7e-08) were significantly associated with diffuse type GC (Figure 2B–2D) and the blue (R=0.22, p=3e-05) and green (R=0.26, p=4e-07) module were also significantly correlated with tumor differentiation.

### Hub genes in blue and green module

**Figure 3:**
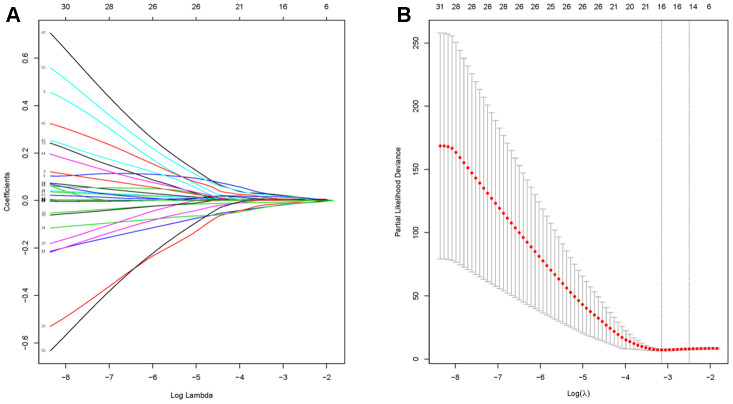
**Identification of hub genes using LASSO regression.** (**A**) The trajectory of each prognosis-related candidate gene’s coefficient in diffuse type GC was observed in the LASSO coefficient profiles with the changing of the lambda in LASSO algorithm. (**B**) After the 10-fold cross-validation, a confidence interval was got for partial likelihood deviance as the lambda changed.

Before conducting the univariate cox analysis, we first adopted the merge function in R to integrate the expression profiles of the 1794 module genes with corresponding 71 diffuse type GC patients’ survival time and status information. 63 overall survival (OS)-related hub genes (p<0.1) were picked out by univariate cox regression analysis. According to the characteristics of variable selection and regularization, while fitting the generalized linear model, LASSO regression was performed to select hub genes for predicting the prognosis of high-performance patients (Figure 3A, 3B). This approach is popular in machine learning and is implemented through the “glmnet” package. 16 hub genes ("RALA", "DDX3Y", "ERP29", "SRSF5", "SLC9A3R1", "FBXO9", "GMDS", "CCNI", "LEF1", "RFX5", "CAST", "ELMO1", "FRZB", "TMEM92", "SELP" and "NMB") were identified.

### Construction of risk score model based on multivariate Cox regression

**Figure 4:**
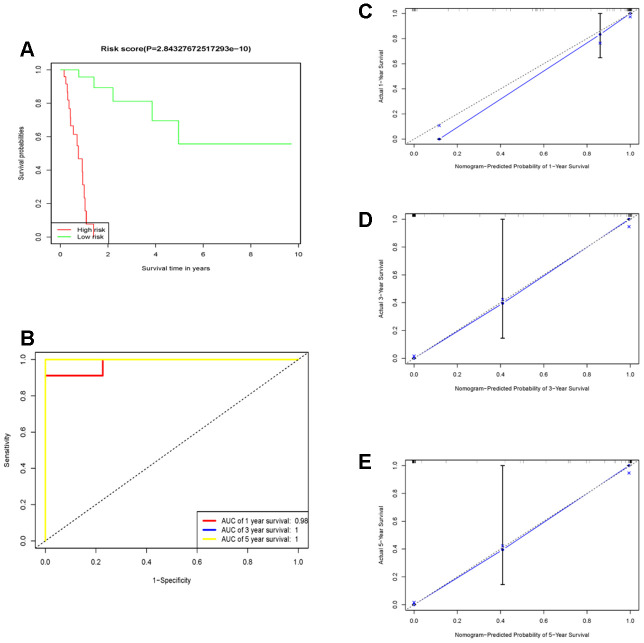
**The prognostic performance of the 10 genes model in the TCGA-STAD.** (**A**) Survival analysis of the high-risk group and the low-risk group using Kaplan–Meier curves. (**B**) The prognostic efficiency of the 10 genes model for survival time. ROC curves of the 10 genes signature for predicting 1-, 3 -and 5- year survival were analyzed. (**C**–**E**) The comparison between predicted and actual outcome for 1-, 3-, and 5-year survival probabilities was showed in the calibration plots.

First, followed by multivariate cox regression, the optimal 10 prognostic signatures in diffuse type GC samples (Table 1), including: “RALA”, “DDX3Y”, “SRSF5”, “SLC9A3R1”, “GMDS”, “LEF1”, “RFX5”, “CAST”, “FRZB” and “SELP”, were picked out. Then, the risk score (RS) model for OS was identified. All 71 diffuse type GC samples were endowed with a RS by RS model calculation and divided into high- and low- RS groups using the median value as the cut-off point. The concordance index (C-index) of this model was 0.97, indicating that this model had quite high reliability. Figure 4A showed that patients in the low-risk group had longer OS (p < 2.8e-10) than those of the high-risk group. The relation of high- and low expression of 10 genes to OS were viewed in Supplementary Figures 2–11. To determine the predictive accuracy of this prognostic model, we performed a receiver operating characteristic (ROC) curve analysis, which demonstrated that the area under the curve (AUC) was 0.98 for 1-year survival, 1 for 3-year survival and 1 for 5-year (Figure 4B). And the calibration curve supported the predicting model of 10 genes (Figure 4C–4E). The survival, 10 genes expression and risk score for TCGA samples could be viewed in Supplementary Table 1.

**Table 1:** The univariate and multivariate Cox regression analysis between 10 markers and OS in diffuse type GC.

	**univariate-cox**				**multivariate-cox**		
	**HR**	**95%CI**	**p-value**		**HR**	**95%CI**	**p-value**
CAST	1.0138	1.0020-1.0260	0.0225		1.0316	1.0116-1.0520	0.0018
DDX3Y	1.019	1.0000-1.0380	0.0474		1.1402	1.0638-1.2220	0.0002
FRZB	1.0124	1.0050-1.0190	0.0005		1.0634	1.0323-1.0955	4.98E-05
GMDS	1.0058	0.9989-1.0130	0.0999		1.0287	1.0111-1.0466	0.0012
LEF1	1.0437	1.0080-1.0800	0.0146		1.1671	1.06421.2797	0.001
RALA	1.0157	0.9972-1.0350	0.0959		1.0769	1.0300-1.1259	0.0011
RFX5	0.9669	0.9420-0.9927	0.012		0.8518	0.7769-0.9339	0.0006
SELP	1.0199	1.0020-1.0380	0.0291		1.0453	1.0115-1.0804	0.0083
SLC9A3R1	1.0023	0.9998-1.0050	0.075		1.0094	1.0007-1.0181	0.0343
SRSF5	0.9891	0.9775-1.0010	0.071		0.8877	0.8333-0.9456	0.0002

Notes: Hazard ratio (HR), 95% confidence intervals (95%CI)

### Validation of 10-genes RS model in external independent cohort

**Figure 5:**
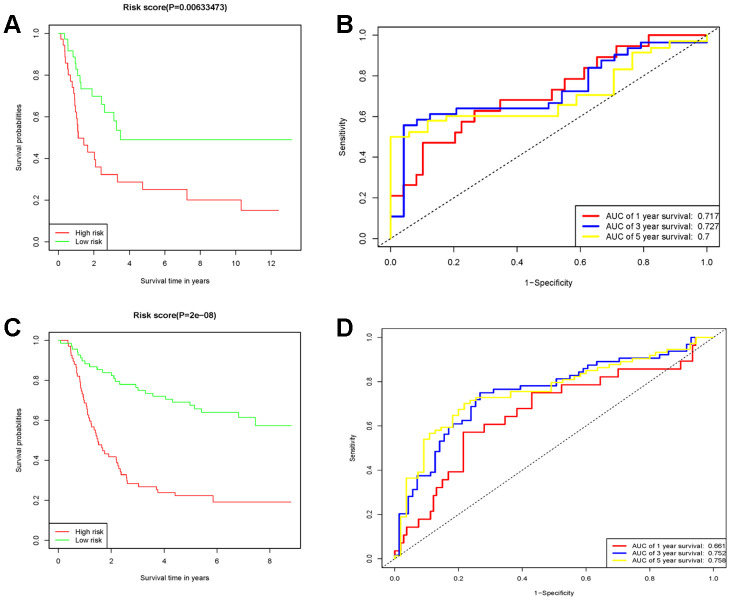
**The prognostic performance of the 10 genes model in the GSE15459 and GSE62254.** (**A**) Survival analysis of the high-risk group and the low-risk group using Kaplan-Meier curves in the GSE15459. (**B**) The prognostic efficiency of the 10 genes model for survival time. ROC curves of the 10 genes signature for predicting 1-, 3 -and 5- year survival were analyzed in the GSE15459. (**C**) Survival analysis of the high-risk group and the low-risk group using Kaplan-Meier curves in the GSE62254. (**D**) The prognostic efficiency of the 10 genes model for survival time. ROC curves of the 10 genes signature for predicting 1-, 3 -and 5- year survival were analyzed in the GSE62254.

75 diffuse type GC patients from GSE15459 were remaining after removing the non-diffuse type and survival time as zero. Consistent with the results in the TCGA cohort, the low RS group had good performance in OS (P=0.00633473) than in the high RS group (Figure 5A). Moreover, the AUCs for 1-year, 3-year and 5-year survival in the validation cohort were 0.717, 0.727 and 0.7 (Figure 5B), respectively. There were 135 diffuse type GC patients in GSE62254 by the above same processing. The low RS group had good performance in OS (P=2E-8) than the high RS group as same as above (Figure 5C). the AUCs for 1-year, 3-year and 5-year survival in the validation cohort were 0.661, 0.752 and 0.758 (Figure 5D). Thus, the RS model had again been proved to be reliable. The survival, 10 genes expression and risk score for GSE15459 and GSE62254 samples could be viewed in Supplementary Tables 2 and 3.

### GSEA

**Figure 6:**
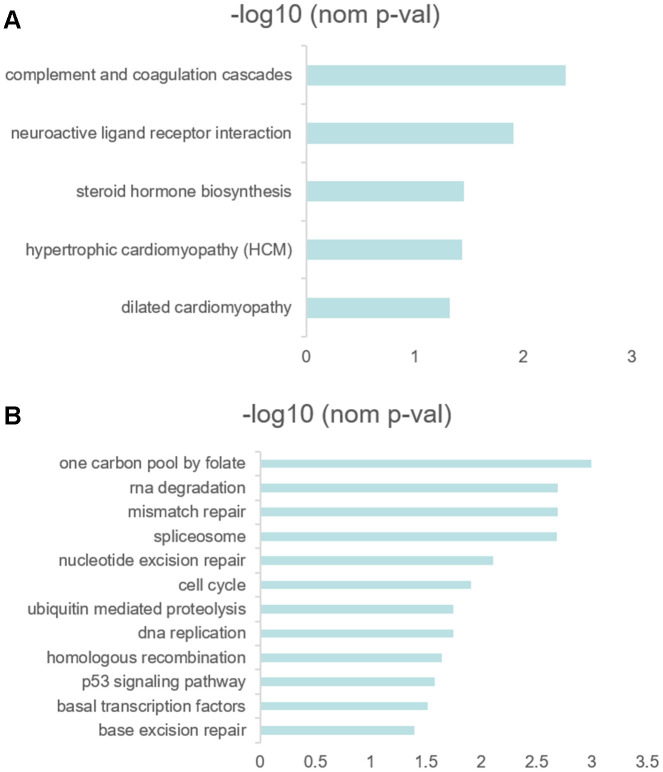
(**A**, **B**) GSEA results revealed the significantly enriched biological processes between two RS levels.

In order to explore the difference of functions and pathways of high- and low- RS groups, the gene set enrichment analysis (GSEA) was performed using the risk score as the reference phenotype. The GSEA analysis revealed that the complement and coagulation cascades, neuroactive ligand-receptor interaction, hypertrophic cardiomyopathy (HCM), steroid hormone biosynthesis and dilated cardiomyopathy were upregulated in the high-risk group (Figure 6A). Then, the spliceosome, one carbon pool by folate, nucleotide excision repair, cell cycle, RNA degradation, mismatch repair, DNA replication, ubiquitin mediated proteolysis, homologous recombination, p53 signaling pathway, basal transcription factors and base excision repair were upregulated in the low-risk group (Figure 6B). To investigate the enrichment pathways of 10 prognostic genes, we divided the samples into two groups, a group samples with the expression of upper quantile for one of 10 prognostic genes and another group samples with the expression of lower quantile for one of 10 prognostic genes. All 10 genes related pathway enrichment could be viewed in Supplementary Table 4.

## DISCUSSION

Gastric cancer is a common malignancy and had poor response to treatment for its strong heterogeneity. Previous studies investigated gastric cancer samples as a whole [9] [10], which made researchers miss some important information and even obtaining imprecise conclusion. As stated above, for Lauren extensively used in clinical practice for the ability of perceiving the histology and biology of GC, diffuse type GC patients usually progressed faster after the diagnosis or were diagnosed in the late stage. Since the significant heterogeneity of two type, it was urgent to find a predicting model for OS of diffuse type GC facilitating clinical decision-making.

WGCNA, the algorithm aiming to investigate the relationships between genes and phenotype of samples, can be applied to identify complex biological mechanisms responsible for the target phenotypes. The unsupervised hierarchical clustering method selected by WGCNA avoided potential biases and subjective decisions attributed to the selection of the candidate genes previously reported associated with diffuse type GC. We applied a system biology approach, namely WGCNA, to analyze TPM expression dataset to identify the networks and genes associated with diffuse type GC. The lasso regression algorithm as the precision and efficiency of variable selection reduced the dimension of model and cox regression algorithm was performed to identify 10 prognostic genes model. The C-index, AUCs and survival difference of high- and low- risk groups well demonstrated perfect performance of predicting survival of model in diffuse type GC. And the similar phenomenon was observed in the independent cohort of GSE15459 and GSE62254.

The green module and blue module were associated with measles, HTLV-I (human T-cell lymphotropic virus type I) infection, Epstein-Barr virus (EB) infection, measles, pathways in cancer, focal adhesion, cell adhesion molecules (CAMs), apoptosis and many immune relevant pathways (the detailed KEGG enrichment can be found in Supplementary Table 5), indicated that not only EB but also HTLV-1 and measles might be related with the occurrence and progression of diffuse type GC [11, 12]. However, the study about HTLV-1 and gastric cancer were few [13, 14]. Matsumoto S et.al [14] drew a conclusion that HTLV-1 infection likely reduced the risk of helicobacter pylori infection and proliferation and, thereby, the risk of gastric cancer. However, helicobacter pylori infection only played an important role in the intestinal type cancer [15]. Reverse effect was possible to be present in the diffuse type GC. Certainly, the real results would need to get in the subsequent investigation. The measles was thought to be relevant to lung cancer, whereas poor evidence supported [16]. Then, immune relevant pathways enrichment was consistent with the previous studies [17–19]. Interestingly, the two hub modules with diffuse type GC were highly correlated with poorly differentiated tumors, that mean diffuse type GC with poor differentiation. The two points proved that the WGCNA approach of identifying genes related with diffuse type GC was enough reliable.

Then, we analyzed the KEGG enrichment between high-and low- RS groups using GSEA. The nucleotide excision repair, cell cycle, RNA degradation, mismatch repair, DNA replication, p53 signaling pathway and base excision repair were upregulated in low RS patients. However, only complement and coagulation cascades, neuroactive ligand receptor interaction, steroid hormone biosynthesis, hypertrophic cardiomyopathy (HCM) and dilated cardiomyopathy (nominal p<0.05) were upregulated in high RS patients. The phenomenon was involved with not enough sample size. The significant enrichment differences may be a classification method for diffuse type GC patients or an idea for identifying the subtype of other malignancies. Thus, we should validate our findings in more sample size in the future. According to GSEA analysis of every hub genes, many pathways associated with tumor were up-regulated or down-regulated between the upper quantile expression group and the lower quantile expression group. But the implied mechanism under these genes needed a large sample size to investigate in the late studies.

Our study has several limitations. There was not enough sample size due to the small percentage of diffuse type GC in all GC. As a retrospective study, the patient cohort was heterogeneous, and the significance and robustness of the results and hub genes in the prognostic assessment should be validated in prospective cohorts.

In conclusion, our study is the first study to screen the characteristic hub genes of diffuse type GC using WGCNA and to construct a prognostic model based on hub genes. The prognostic predictive model of 10 genes was proved to be able to accurately investigate the prognosis of diffuse type GC. This model might be applied to identify the high-risk patients, and assess the prognosis, so as to facilitate the precise treatment in diffuse type GC.

## MATERIALS AND METHODS

### Data collection and processing

Public gene-expression data and full clinical annotation were searched in the Gene-Expression Omnibus (GEO) and TCGA database. The procedure used for data set selection in the GEO database was as follows. The following search parameters were used: (cancer) OR tumor) OR carcinoma) OR adenocarcinoma) AND ((gastric) OR Stomach)) AND "Homo sapiens"[porgn: txid9606]. In the initial search, 755 items were recognized. The eligible criteria included that: 1) owning Lauren classification; 2) owning survival information and 3) at least 50 diffuse type gastric cancer. We removed the datasets that don’t meet the criteria by checking them one by one carefully. In total, we gathered three patient cohorts with gastric cancer for this study: TCGA-STAD, GSE62254 and GSE15459.

The TCGA-STAD RNA-seq data and clinical data of the 375 GC samples were downloaded by using the “TCGAbiolinks” package in R [20], which was used as a training set for prognostic prediction of the multi-gene signature. The RNA-seq data for 19505 genes measured as fragments per kilobase of transcript per million mapped reads (FPKM), which were converted to transcripts per million (TPM) after removing duplicated genes and zero expression genes [21]. We obtained the raw expression and clinical data of GC samples from GSE622254 and GSE15459 via GEO database which was used as a validation set. The raw expression data was processed by log10-transformed. The demographics are listed in Table 2.

**Table 2:** Basic characteristics of the datasets.

**Variables**	**TCGA**	**GSE62254**	**GSE15459**
	**n=71**	**n=135**	**n=75**
**Age (Mean** ± **SD)**	62.25±10.47	58.58±12.54	59.87±13.20
**OS(Mean ± SD)**	1.23±0.75	3.87±2.73	3.18±3.67
**Gender**			
Male	46(64.79%)	75(55.56%)	36(48.00%)
Female	25(35.21%)	60(44.44%)	39(52.00%)
**Status**			
Alive	50(70.42%)	56(41.48%)	34(45.33%)
Dead	21(29.58%)	79(58.52%)	41(54.67%)
**Stage**			
I	9(12.67%)	5(3.70%)	9(12.00%)
II	20(28.17%)	35(25.93%)	12(16.00%)
III	32(45.07%)	49(36.30%)	31(41.33%)
IV	6(8.45%)	46(34.07%)	23(30.67%)
Unknown	4(5.63%)	0(0%)	0(0%)
**T category**			
T1	0(0%)	0(0%)	-
T2	19(26.76%)	65(48.15%)	-
T3	27(38.03%)	60(44.44%)	-
T4	25(35.21%)	10(7.40%)	-
Unknown	0(0%)	0(0%)	-
**N category**			
N0	17(23.94%)	8(5.93%)	-
N1	22(30.98%)	54(40.00%)	-
N2	14(19.72%)	41(30.37%)	-
N3	17(23.94%)	32(23.70%)	-
Unknown	1(1.41%)	0(0%)	-
**Grade**			
G1	2(2.82%)	-	-
G2	1(1.41%)	-	-
G3	64(90.14%)	-	-
G4	0(0%)	-	-
GX	4(5.63%)	-	-

### Weighted Gene co-expression network construction

To find modules of highly correlated with diffuse type GC, WGCNA was performed using the WGCNA R package [22] and carried out on top 50% most variable genes (9752 genes). An unsupervised co-expression relationship was initially built on the basis of the adjacency matrix of connection strengths by using Pearson’s correlation coefficients for gene pairs. This matrix was increased to β = 4 based on the scale-free topology criterion. The adjacency matrix of gene expression data for GC patients was then clustered using topological overlap matrix analysis. Finally, the dynamic tree cut algorithm was applied to the dendrogram for module identification with the mini-size of module gene numbers set as 30 and a cut height of 0.95. The module eigengenes (MEs) as the first principal component was performed with the expression data for each co-expressed module in all GC samples. The module that had the strongest association with diffuse type GC was selected for further analysis. The WGCNA algorithm was described in detail by Zhang Bin et al. [23].

### The construction of multi-gene signature risk score model

To identify the prognostic genes in the module correlation with diffuse type GC, the univariate Cox regression analysis was applied using “survival” package. The genes with P<0.1 were defined to be related to the over survival. Given the already detected prognostic genes, we further investigated the significant signature associated with survival across the diffuse type GC samples by the LASSO regression model using “glmnet” package. Then, multivariate Cox regression analysis was performed with the “survival” package to screen out independent prognostic factors from these robust markers, which were conducted the risk score model: prognostic score =∑(C×EXP_mRNA_), where EXP is the TPM value of the gene and C is the regression coefficient for the corresponding gene in multivariate Cox hazard model analysis. ROC plot with AUC and calibration curve were derived to assess predictive significance of the model using “rms”, “survival” and “timeROC” package in R. Additionally, survival time difference between high- and low-risk levels were estimated via Kaplan-Meier analysis. The risk score model was validated with an independent data set GSE15459 and GSE62254.

### Functional pathway analysis

To investigate the biological function of different risk score groups, we further conducted GSEA using the risk score as the phenotype [24]. With the GSEA 4.0.3 software via the Java platform, we derived the “c2.cp.kegg.v7.1.symbols.gmt gene sets” as the reference set. We divided the samples into two groups, a group samples with the expression of upper quantile for one of 10 prognostic genes and another group samples with the expression of lower quantile for one of 10 prognostic genes. The enriched signaling pathways with FDR < 0.25 or nominal p < 0.05 were defined as statistically significant.

## Supplementary Material

Supplementary Figures

Supplementary Table 1

Supplementary Table 2

Supplementary Table 3

Supplementary Table 4

Supplementary Table 5
