# Supplementary material for: Identification of subtype-specific genes signature by WGCNA for prognostic prediction in diffuse type gastric cancer
Source: Aging (Albany NY). 2020 Sep 11;12(17):17418–35. doi: 10.18632/aging.103743 (PMC7521533; doi:10.18632/aging.103743)
Supplement: Supplementary Figures [file aging-12-103743-s006..pdf]

SUPPLEMENTARY FIGURES

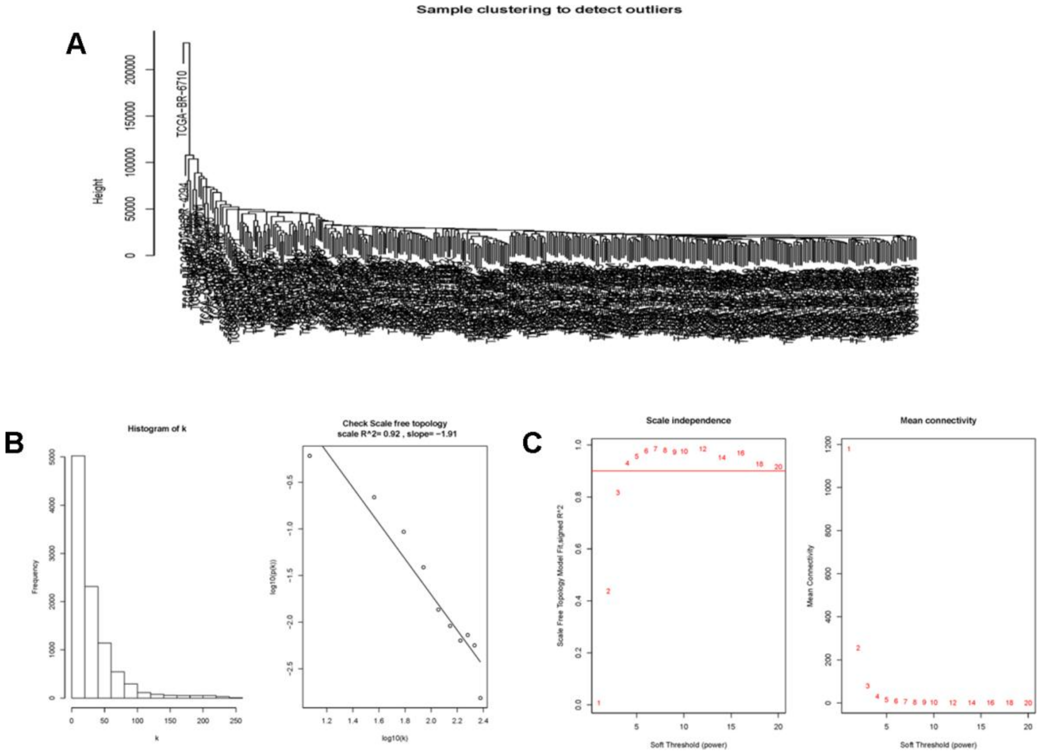

Supplementary Figure 1. (**A**) Sample clustering to detect outliers. (**B**, **C**) Determination of soft-thresholding power in the co-expression network analysis.

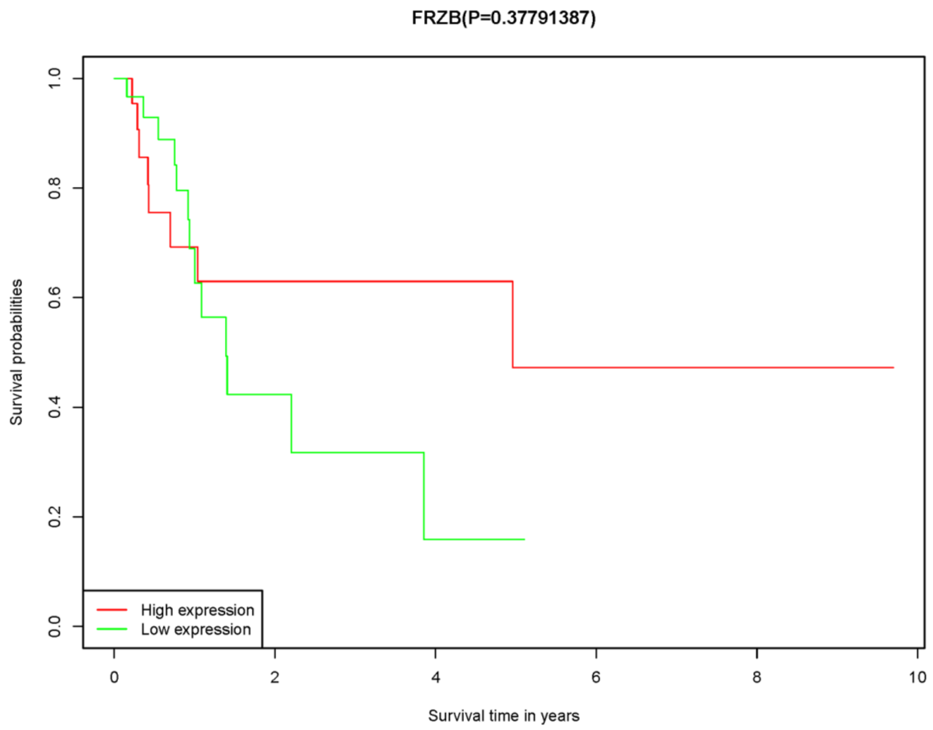

Supplementary Figure 2. The relation of high- and low expression of 10 hub genes for OS.

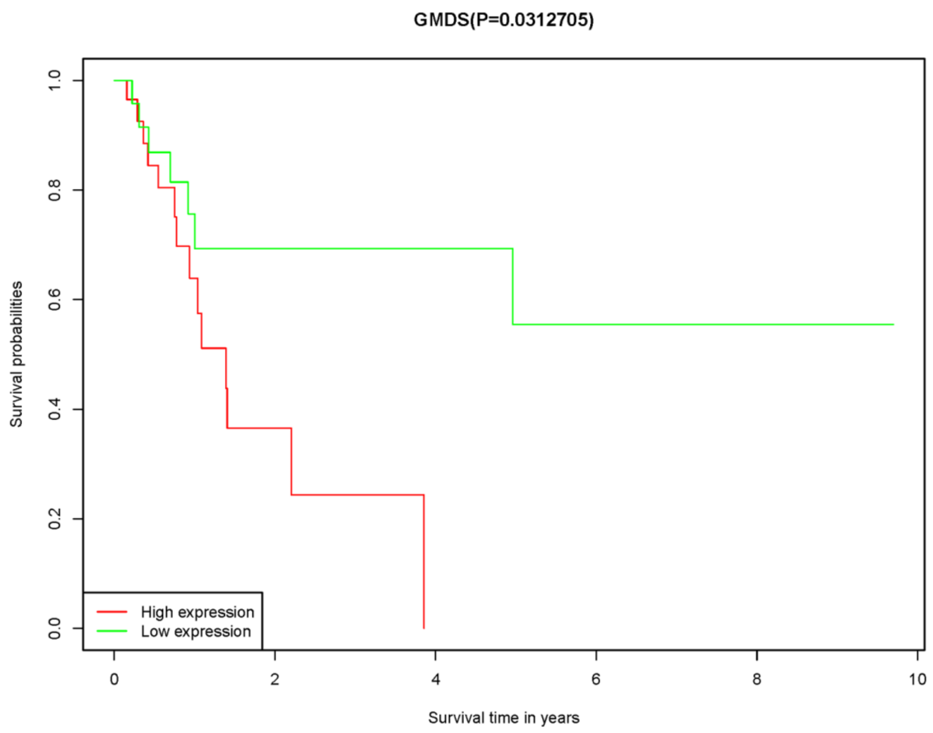

Supplementary Figure 3. The relation of high- and low expression of 10 hub genes for OS.

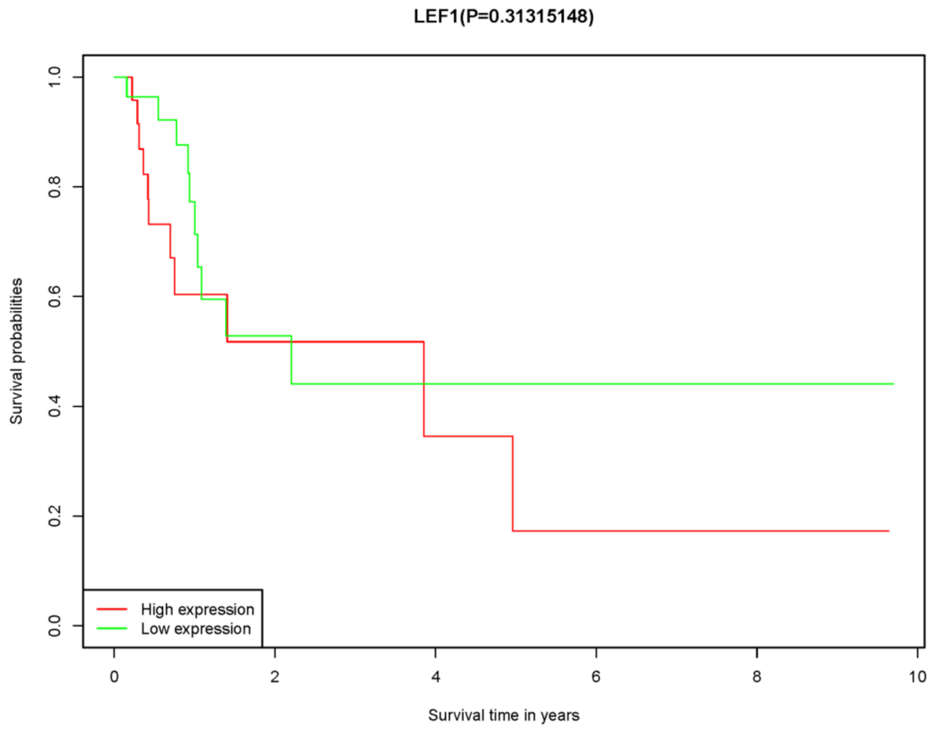

Supplementary Figure 4. The relation of high- and low expression of 10 hub genes for OS.

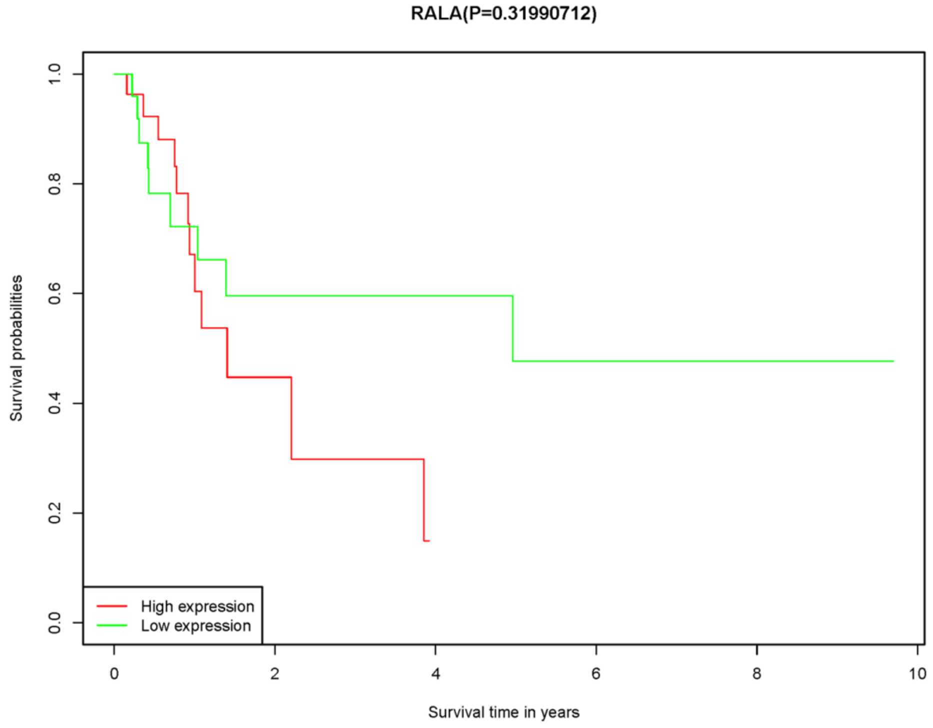

Supplementary Figure 5. The relation of high- and low expression of 10 hub genes for OS.

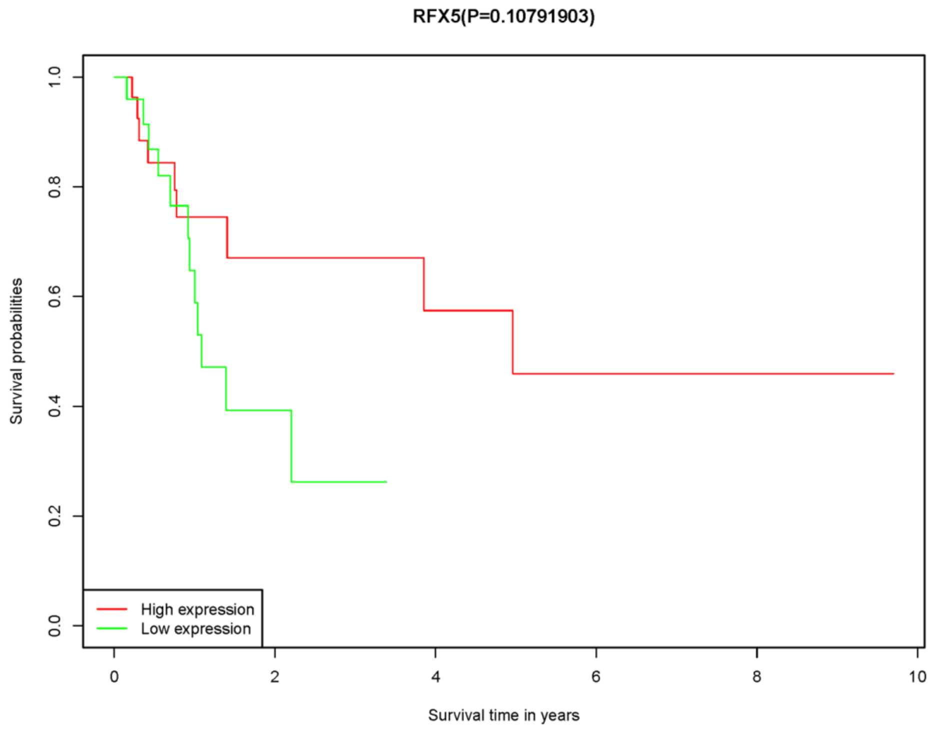

Supplementary Figure 6. The relation of high- and low expression of 10 hub genes for OS.

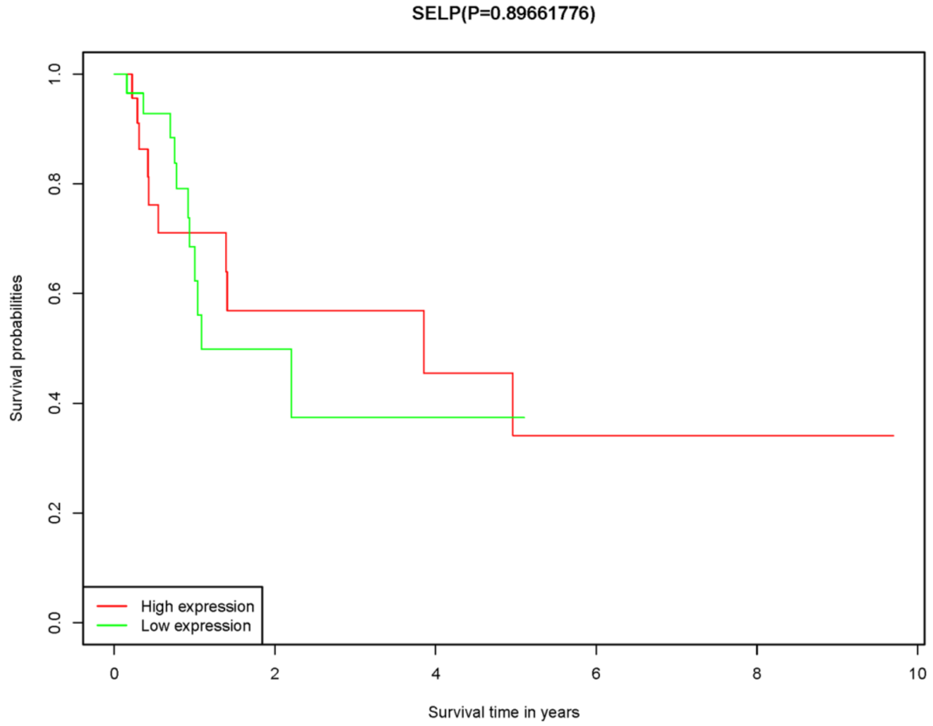

Supplementary Figure 7. The relation of high- and low expression of 10 hub genes for OS.

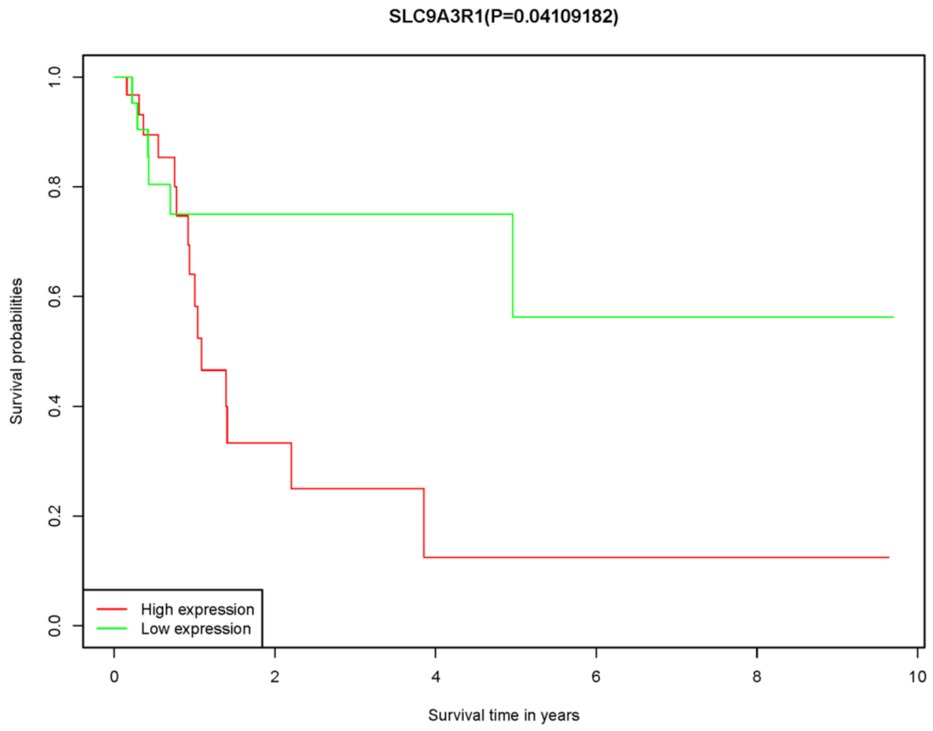

Supplementary Figure 8. The relation of high- and low expression of 10 hub genes for OS.

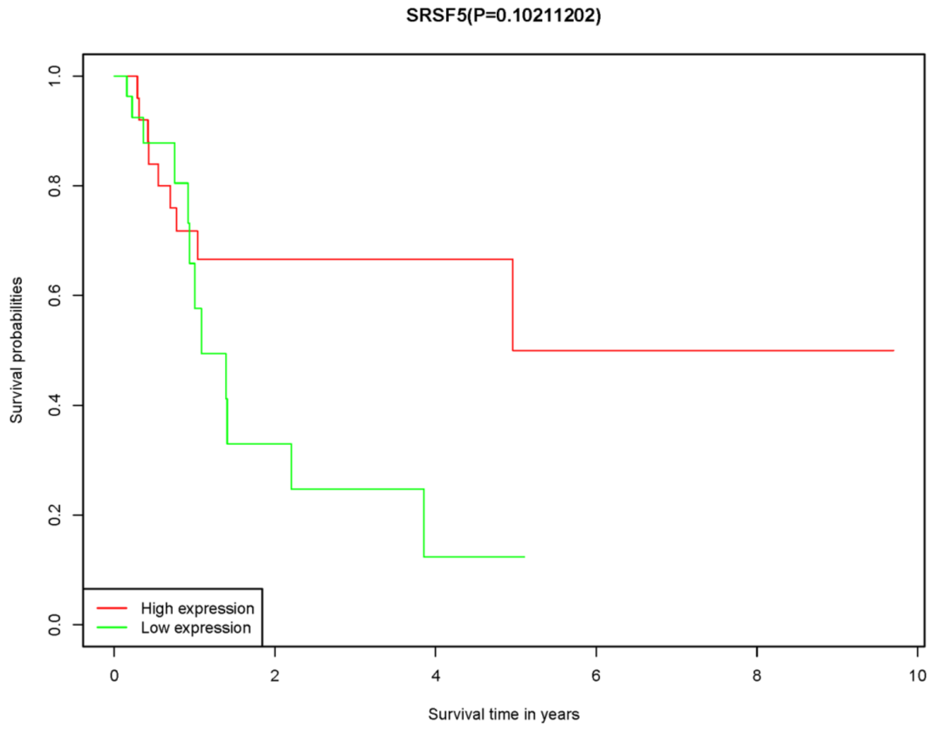

Supplementary Figure 9. The relation of high- and low expression of 10 hub genes for OS.

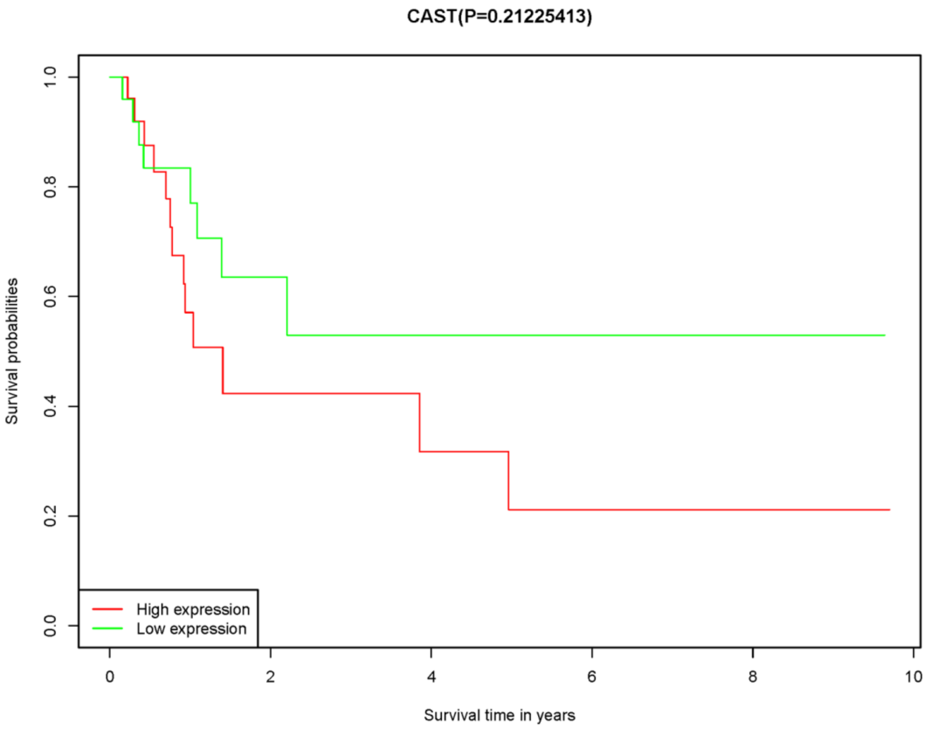

Supplementary Figure 10. The relation of high- and low expression of 10 hub genes for OS.

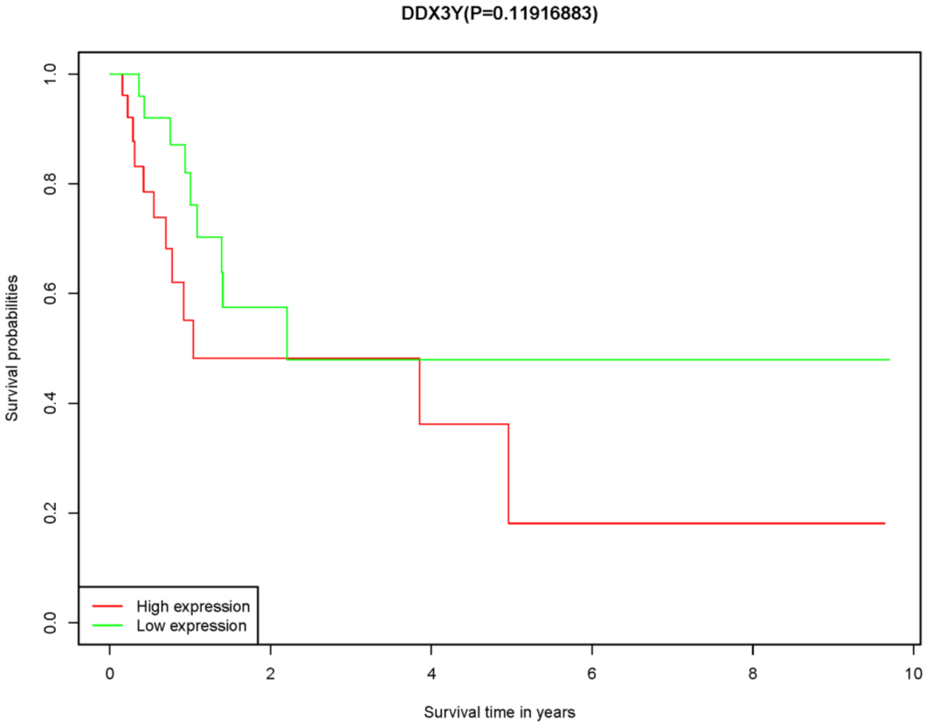

Supplementary Figure 11. The relation of high- and low expression of 10 hub genes for OS.
